# Determinants of Long-Term Mortality in Patients with Acute Coronary Syndromes Requiring CICU Admission: Diagnosis Versus Vulnerability in a Contemporary Real-World Cohort

**DOI:** 10.3390/life16040586

**Published:** 2026-04-01

**Authors:** Aneta I. Gziut-Rudkowska, Adam Kern, Krystian Bojko, Robert J. Gil, Jacek Bil

**Affiliations:** 1 Department of Cardiology, National Medical Institute of the Ministry of Interior and Administration, 02-507 Warsaw, Poland; aneta.gziut@pimmswia.gov.pl (A.I.G.-R.); robert.gil@pimmswia.gov.pl (R.J.G.); 2 Department of Cardiology and Internal Medicine, School of Medicine, Collegium Medicum, University of Warmia and Mazury in Olsztyn, 10-082 Olsztyn, Poland; adam.kern@uwm.edu.pl (A.K.); krystian.bojko@uwm.edu.pl (K.B.)

**Keywords:** cardiac intensive care, acute coronary syndromes, long-term prognosis, frailty, multimorbidity, atrial fibrillation

## Abstract

**Background:** Contemporary cardiac intensive care units (CICUs) increasingly care for elderly and multimorbid patients, yet the relative contribution of admission diagnosis versus underlying comorbidity burden to long-term prognosis remains unclear. We aimed to identify independent determinants of 5-year mortality following emergent CICU admission, with particular focus on acute coronary syndromes (ACS). **Methods:** We retrospectively analyzed 1299 consecutive adults admitted emergently to a tertiary CICU between 2017 and 2019. Baseline characteristics, comorbidities, and in-hospital management were assessed. Five-year follow-up was available for 825 patients with ACS. Survival was evaluated using Kaplan–Meier analysis and Cox proportional hazards models to identify independent predictors of all-cause mortality. **Results:** ACS accounted for 63.3% of admissions. In-hospital mortality was 8.0%, without sex-related differences. At 5 years, cumulative mortality among ACS patients was 25.9% and differed across subtypes, being highest in non-ST elevation myocardial infarction (NSTEMI, 31.1%) compared with unstable angina (24.0%) and ST elevation myocardial infarction (STEMI, 18.4%) (*p* < 0.001). However, after multivariable adjustment, ACS subtype and sex were not independently associated with mortality. Instead, older age (HR per year increase), atrial fibrillation, and prior stroke and Charlson Comorbidity Index emerged as the strongest predictors of death. **Conclusions:** In a contemporary, real-world CICU cohort, long-term survival was determined predominantly by age and comorbidity burden rather than by ACS phenotype or sex. These findings support a shift from diagnosis-centered to risk-profile-based long-term stratification following emergent intensive cardiac care.

## 1. Introduction

Cardiac intensive care units (CICUs) have evolved substantially over the past decades. Originally designed as coronary care wards focused primarily on malignant arrhythmias in acute myocardial infarction (MI), modern CICUs have become multidisciplinary environments managing cardiogenic shock, advanced heart failure, complex arrhythmias, structural heart disease, and multiorgan dysfunction. This transformation has been accompanied by shifts in staffing models, procedural intensity, and patient complexity. Contemporary registries illustrate the heterogeneity of case mix: acute coronary syndromes (ACS) account for approximately one-third of CICU admissions, heart failure for nearly one-fifth, and the relative contribution of individual diagnoses varies widely across centers, reflecting differences in triage policies and healthcare systems [1,2,3]. These changes underscore the need for updated characterization of patient profiles and long-term outcomes in contemporary CICU populations.

Beyond the index hospitalization, survivorship after critical cardiovascular illness is associated with substantial long-term risk. Studies in general intensive care unit cohorts have demonstrated persistently elevated mortality and functional decline extending years after discharge. However, data specific to CICU populations—particularly with extended follow-up—remain limited. Importantly, the modern CICU population is increasingly older and burdened with multimorbidity, including atrial fibrillation, chronic kidney disease, prior cerebrovascular disease, and malignancy. In such patients, biological vulnerability and comorbidity clustering may outweigh the prognostic impact of the index admission diagnosis. Whether long-term outcomes are primarily driven by the acute presentation (e.g., ACS subtype) or by underlying comorbidity burden remains insufficiently clarified. Sex differences in critical cardiovascular care have also attracted increasing attention. Observational studies have reported differences in invasive management strategies and short-term outcomes between women and men in cardiogenic shock and ACS. However, it remains uncertain whether such disparities translate into differential long-term prognosis following emergent CICU admission, particularly when adjusted for age and baseline risk profile [4,5,6,7,8,9].

To address these knowledge gaps, we assembled a consecutive cohort of 1299 adults emergently admitted to a tertiary CICU and characterized their clinical presentation, comorbidity burden, and in-hospital management. Because standardized definitions and complete long-term follow-up were available for patients admitted with ACS—a common indication for CICU admission—we prespecified 5-year outcome analyses within this subgroup. This approach enabled consistent endpoint adjudication while preserving the real-world nature of the overall cohort [10].

The primary aim of this study was to identify independent determinants of 5-year all-cause mortality following emergent CICU admission, with particular emphasis on the relative contribution of age, multimorbidity, admission diagnosis, and sex.

## 2. Materials and Methods

### 2.1. Study Population

This retrospective, single-center observational study was conducted at the CICU of the National Medical Institute of the Ministry of Interior and Administration in Warsaw, Poland, a tertiary referral cardiovascular center providing 24 h invasive cardiology services, including primary percutaneous coronary intervention (PCI), advanced heart failure management, and structural heart interventions.

The CICU is a cardiologist-led, closed unit comprising 21 monitored beds, admitting adult patients requiring intensive cardiovascular monitoring or advanced therapeutic support. Admission criteria include ACS, cardiogenic shock, acute or decompensated heart failure, life-threatening arrhythmias, pulmonary embolism with hemodynamic instability, severe valvular disease requiring urgent evaluation, and other acute cardiovascular conditions necessitating invasive monitoring or high-dependency care.

All consecutive adult patients admitted emergently to the CICU between January 2017 and December 2019 were screened and included, irrespective of admission diagnosis. Elective admissions were excluded. This approach ensured a comprehensive and unselected cohort reflecting real-world CICU case mix and contemporary practice patterns. The study consisted of two complementary analytical components. First, in-hospital outcomes were assessed in the entire cohort of patients admitted to the CICU. Second, long-term (5-year) survival analysis was performed in a predefined subgroup of patients with ACS, for whom complete and systematically verified follow-up data were available. This approach allowed for a comprehensive evaluation of early outcomes in the overall population, while ensuring methodological consistency and data completeness in long-term analyses.

### 2.2. Data Collection

Clinical data were obtained retrospectively from institutional electronic medical records and hospital databases using standardized extraction protocols. Baseline variables included demographic characteristics (age, sex), cardiovascular risk factors, prior cardiovascular history, and relevant non-cardiovascular comorbidities (including chronic kidney disease, prior stroke, malignancy, and dementia).

The primary reason for CICU admission was determined based on the final discharge diagnosis and categorized according to predefined clinical groups. For patients with acute coronary syndromes, diagnoses were established according to the Fourth Universal Definition of Myocardial Infarction, and high-sensitivity cardiac troponin assays were routinely used during the study period.

In-hospital management variables included the use of invasive coronary angiography, PCI, coronary artery bypass grafting (CABG), and other relevant therapeutic strategies. Data regarding left ventricular ejection fraction (LVEF), occurrence of in-hospital complications, and supportive therapies were also collected.

Discharge pharmacotherapy, including antiplatelet therapy, statins, beta-blockers, and renin–angiotensin system inhibitors, was recorded and included in the multivariable Cox proportional hazards model to account for the potential impact of optimal medical therapy on long-term outcomes.

Short-term outcomes were defined as in-hospital mortality and clinical status at discharge. All data were anonymized prior to analysis.

Additionally, to provide a standardized assessment of comorbidity burden as a surrogate of baseline clinical vulnerability, the Charlson Comorbidity Index (CCI) was calculated for each patient [11]. The CCI assigns weighted points to predefined comorbid conditions (e.g., heart failure, diabetes, prior stroke, chronic kidney disease, malignancy), with higher scores reflecting greater overall disease burden and worse prognosis. In addition, age-adjusted CCI was derived by incorporating additional points based on age categories. Comorbidities included in the index were identified based on documented medical history at baseline. The CCI was calculated according to the original method described by Charlson et al., with established weighting of individual conditions. When appropriate, clinically relevant categories available in the dataset were mapped to corresponding CCI components. The index was subsequently used as a composite measure of comorbidity burden in statistical analyses.

### 2.3. Subgroup of Acute Coronary Syndrome

Within the overall CICU cohort, a predefined subgroup of patients admitted with ACS was identified for detailed long-term outcome analysis. ACS diagnoses (ST-elevation myocardial infarction [STEMI], non-ST elevation myocardial infarction [NSTEMI], unstable angina) were established according to the Fourth Universal Definition of Myocardial Infarction. Cardiac troponin assays were routinely used during the study period as the standard biomarker for myocardial injury detection. Unstable angina was diagnosed in patients presenting with ischemic symptoms without dynamic troponin elevation and without objective evidence of myocardial necrosis.

Patients with ACS were selected for extended follow-up because of the standardized diagnostic criteria, uniform management algorithms, and availability of complete outcome data. Within this subgroup, additional clinical and procedural variables were collected to enable granular assessment of disease severity and treatment strategies. These included angiographic extent of coronary artery disease (single- vs. multi-vessel involvement), performance and timing of coronary angiography, type of revascularization (PCI, CABG or conservative management), and completeness of revascularization when applicable.

This focused data collection allowed for integrated evaluation of clinical presentation, management patterns, and long-term outcomes in a well-defined, high-risk CICU population.

### 2.4. Follow-Up

Patients admitted with ACS were followed for up to five years after the index hospitalization. The primary long-term endpoint was all-cause mortality. Mortality status was obtained from national civil registry records, which capture deaths occurring throughout the country, ensuring complete ascertainment of survival status for all patients included in the ACS subgroup. Consequently, no patients were lost to follow-up for the primary endpoint of all-cause mortality.

Information regarding non-fatal events—including recurrent MI, ischemic or hemorrhagic stroke, and unplanned cardiovascular rehospitalizations—was obtained from institutional electronic medical records and regional hospital databases. Events occurring outside the national healthcare system could not be systematically verified.

The follow-up period was calculated from the date of index CICU admission to the date of death or completion of 5-year follow-up, whichever occurred first.

### 2.5. Statistical Analysis

Continuous variables are presented as mean ± standard deviation (SD) or median with interquartile range (IQR), depending on distribution, and were compared using Student's *t*-test or Mann–Whitney U test, as appropriate. Categorical variables are expressed as counts and percentages and were compared using the χ^2^ test or Fisher’s exact test.

Survival analyses were performed using the Kaplan–Meier method, with between-group differences assessed using the log-rank test. Cox proportional hazards regression models were constructed to identify independent predictors of 5-year all-cause mortality within the ACS subgroup.

The CCI was summarized as a continuous variable (mean ± SD) in the baseline characteristics to provide a descriptive overview of comorbidity burden in the study population. For survival analyses, CCI was modeled as a categorical variable (≤2, 3–4, ≥5) to enhance clinical interpretability and to avoid assumptions of linearity of risk across the full range of CCI values.

To minimize redundancy and collinearity, two modeling strategies were considered: (1) inclusion of the CCI as a global comorbidity measure, and (2) inclusion of selected individual comorbid conditions. Given that several variables (e.g., prior stroke, heart failure, diabetes, and malignancy) are components of the CCI, these variables were not entered simultaneously with CCI in the final model to avoid overadjustment and structural collinearity. Accordingly, CCI was retained as an integrated measure of overall comorbidity burden, while clinically relevant variables not captured by the index (e.g., atrial fibrillation) were included separately.

Variables associated with mortality at *p* < 0.10 in univariable analysis and clinically relevant covariates were considered for inclusion in the multivariable model. Residual collinearity among candidate variables was assessed using variance inflation factors (VIF), and variables demonstrating significant multicollinearity were excluded or modeled separately. The proportional hazards assumption was evaluated using Schoenfeld residuals.

Sensitivity analyses were performed to assess the robustness of the findings by comparing models including CCI versus models including individual comorbidities. Consistency of effect estimates across these approaches supported the stability of the identified predictors.

Subgroup analyses were performed according to sex. A two-sided *p*-value < 0.05 was considered statistically significant. Statistical analyses were conducted using R software v. 4.5.1. (R Foundation for Statistical Computing, Vienna, Austria).

## 3. Results

### 3.1. Population Characteristics

**Figure 1:**
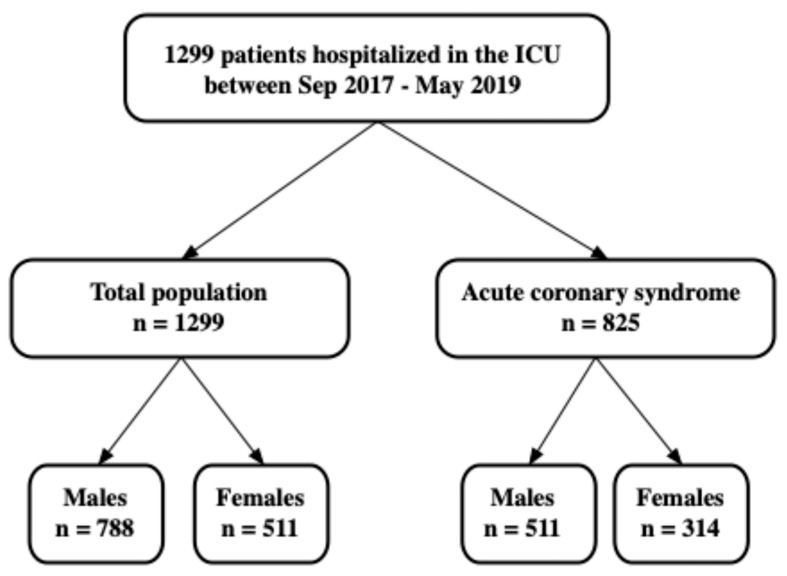
Study flowchart.

A total of 1299 patients (511 women [39.3%] and 788 men [60.7%]) were admitted to the CICU. Of these, the majority (N = 825, 63.3%) were admitted with ACS (Figure 1).

The cohort was characterized by a high burden of cardiovascular risk factors and comorbidities. Arterial hypertension was present in 72.8% of patients, diabetes in 27.1%, established coronary artery disease in 44.3%, and chronic kidney disease in 15.5%. A history of heart failure was documented in 21.5%. Prior coronary revascularization was common, with previous PCI in 21.6% and prior CABG in 5.8%.

Sex-related differences were observed in baseline comorbidity profiles. Men more frequently had a history of prior PCI (25.3% vs. 16.2%, *p* < 0.001) and CABG (7.9% vs. 2.7%, *p* < 0.001), reflecting a higher prevalence of prior coronary interventions. In contrast, women were more likely to present with atrial fibrillation (25.8% vs. 18.8%, *p* = 0.003), dementia (7.4% vs. 3.2%, *p* = 0.001), and malignancy (17.4% vs. 13.1%, *p* = 0.037). The prevalence of chronic kidney disease and prior stroke did not differ significantly between sexes (Table 1).

ACS was the leading reason for CICU admission (63.3%), with similar proportions in women and men (61.4% vs. 64.8%, *p* = 0.216). Within the ACS subgroup, the distribution of STEMI (17.0% vs. 19.3%), NSTEMI (27.8% vs. 32.2%), and unstable angina (17.2% vs. 13.7%) did not differ significantly by sex. Other admission diagnoses—including cardiac arrhythmias, pulmonary embolism, myocarditis, and acute or decompensated heart failure—were similarly distributed between women and men. Aortic stenosis (2.2% vs. 0.6%, *p* = 0.020) and pulmonary edema (4.7% vs. 2.5%, *p* = 0.041) were more frequently observed in women. Remaining admission categories were evenly distributed (Table 2).

### 3.2. Short-Term Outcomes

During the index hospitalization, 66.7% of patients underwent coronary angiography. Men were more likely than women to receive invasive diagnostic evaluation (70.2% vs. 62.0%, *p* = 0.003). PCI was performed in 51.2% of the overall cohort and was significantly more frequent among men compared with women (57.9% vs. 41.3%, *p* < 0.001). In contrast, the rates of CABG were similar between sexes (8.0% overall; 8.2% in women vs. 7.9% in men, *p* = 0.835).

Among patients with myocardial infarction, 27 cases (4.3%) were classified as myocardial infarction with non-obstructive coronary arteries (MINOCA). In the unstable angina subgroup, 31 patients (15.8%) were categorized as having ischemia with non-obstructive coronary arteries (INOCA).

Overall in-hospital mortality was 8.0%, without significant differences between women and men (8.2% vs. 7.9%, *p* = 0.835). Reduced left ventricular systolic function at discharge (ejection fraction < 45%) was observed in 36.4% of patients and did not differ by sex (36.0% vs. 36.9%, *p* = 0.768).

When stratified by primary admission diagnosis, in-hospital mortality varied substantially across groups. The highest short-term mortality was observed in patients admitted with acute or decompensated chronic heart failure (18.5%) and STEMI (13.8%). In contrast, unstable angina and myocarditis were associated with the lowest in-hospital mortality (2.6% each). Pulmonary edema was also associated with elevated in-hospital mortality (15.9%), although this did not reach statistical significance (*p* = 0.079). Both STEMI and acute heart failure were independently associated with significantly worse in-hospital survival compared with other admission categories (*p* = 0.001 for both) (Table 3).

At discharge, patients with ACS (N = 825) received guideline-directed medical therapy with high adherence rates. Acetylsalicylic acid was prescribed in 814 patients (98.7%), and all patients (100%) received a P2Y12 inhibitor as part of dual antiplatelet therapy. Oral anticoagulation was less frequent, with vitamin K antagonists used in 21 patients (2.5%) and non-vitamin K antagonist oral anticoagulants in 60 patients (7.3%). Among cardiovascular medications, beta-blockers were prescribed in 754 patients (91.4%), angiotensin-converting enzyme inhibitors (ACE inhibitors) in 723 patients (87.6%), and angiotensin receptor blockers (ARBs) in 77 patients (9.3%). Mineralocorticoid receptor antagonists were used in 191 patients (23.2%), calcium channel blockers in 139 patients (16.9%), and diuretics in 377 patients (45.7%). Lipid-lowering therapy was widely implemented, with statins prescribed in 817 patients (99%) and fibrates in 8 patients (1.0%). Glucose-lowering therapies included sodium–glucose cotransporter-2 inhibitors (SGLT2 inhibitors) in 27 patients (3.3%), insulin in 52 patients (6.3%), and other oral antidiabetic agents (excluding SGLT2 inhibitors) in 192 patients (23.3%). Trimetazidine was prescribed in 2 patients (0.3%).

### 3.3. Acute Coronary Syndrome 5-Year Outcomes

Among patients admitted with ACS, 5-year survival status was available for 98.9% of individuals. Nine patients (1.1%) were lost to follow-up and were censored at the date of last documented contact.

At 5-year follow-up, overall mortality differed across ACS subtypes (Table 4). Cumulative all-cause mortality was highest among patients with NSTEMI (31.1%), followed by unstable angina (24.0%) and STEMI (18.4%), with a statistically significant difference between groups (*p* < 0.001). Post hoc pairwise comparisons with Bonferroni correction demonstrated a significantly higher risk of death in NSTEMI compared with STEMI (*p* = 0.0003), whereas differences between NSTEMI and unstable angina or between STEMI and unstable angina were not statistically significant.

Rates of unplanned cardiovascular hospitalization, recurrent myocardial infarction, heart failure, and stroke did not differ significantly among ACS subtypes (all *p* > 0.3).

**Figure 2:**
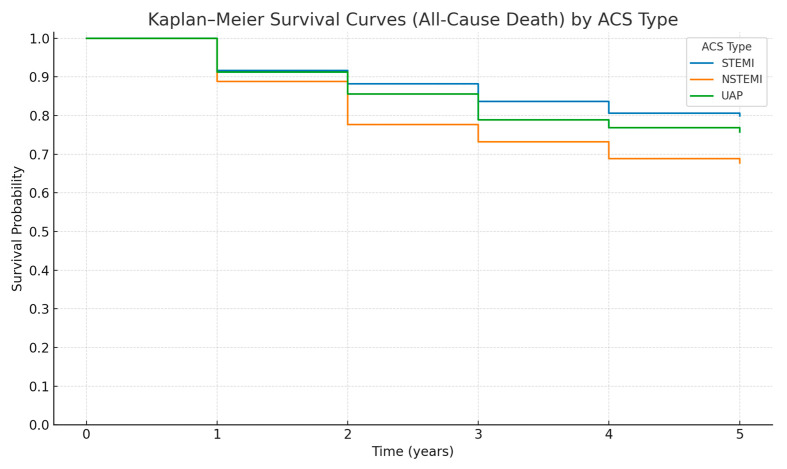
Kaplan–Meier Survival Curves for All-Cause Mortality by Acute Coronary Syndrome Type.

Kaplan–Meier survival analysis confirmed significant divergence in cumulative all-cause mortality across ACS subtypes over the 5-year period (Figure 2). In a Cox proportional hazards model using NSTEMI as the reference category, STEMI was associated with a significantly lower hazard of death (HR 0.59, 95% CI 0.44–0.80, *p* < 0.001). Unstable angina was also associated with a lower hazard compared with NSTEMI (HR 0.72, 95% CI 0.52–1.00), although this did not reach conventional statistical significance (*p* = 0.053).

These findings indicate heterogeneity in long-term mortality risk across ACS subtypes, with NSTEMI patients demonstrating the highest cumulative mortality over five years.

### 3.4. ACS Prognostic Factors for 5-Year Survival

To identify independent predictors of 5-year all-cause mortality, a multivariable Cox proportional hazards regression model was constructed. Variables associated with mortality in univariable analysis (Supplementary Table S1) and clinically relevant covariates were considered for inclusion in the final model.

In the multivariable Cox proportional hazards model, increasing age was independently associated with higher 5-year mortality (HR 1.05 per year, 95% CI 1.03–1.07; *p* < 0.001). Among clinical variables, atrial fibrillation was associated with a significantly increased risk of death (HR 1.65, 95% CI 1.08–2.53; *p* = 0.021), while a history of prior stroke remained a strong predictor of adverse outcome (HR 3.12, 95% CI 1.54–6.31; *p* = 0.002). Dementia was associated with a numerically increased risk; however, this did not reach statistical significance (HR 1.74, 95% CI 0.57–5.28; *p* = 0.329). Importantly, a high comorbidity burden, defined as CCI ≥5, was independently associated with increased long-term mortality (HR 1.88, 95% CI 1.24–2.86; *p* = 0.003) (Table 5).

Importantly, ACS subtype (STEMI, NSTEMI, unstable angina) did not remain independently associated with 5-year mortality after adjustment for age and comorbidity burden. While unadjusted analyses demonstrated lower mortality in STEMI and unstable angina compared with NSTEMI, these associations were attenuated and no longer statistically significant in the multivariable model (all *p* > 0.25).

These findings indicate that long-term prognosis following emergent CICU admission for ACS is primarily driven by patient-related factors, particularly age and pre-existing comorbidities, rather than by the index ACS subtype itself.

## 4. Discussion

This study provides a contemporary, real-world evaluation of long-term outcomes following emergent CICU admission in a large, consecutive cohort. The principal finding is that although unadjusted 5-year mortality differed across ACS subtypes, these differences were no longer significant after accounting for age and comorbidity burden. Instead, long-term survival was primarily determined by patient-related factors, particularly advanced age, atrial fibrillation, and prior stroke.

These findings reinforce the evolving understanding of modern CICU populations. Historically centered on STEMI and malignant arrhythmias, CICUs now increasingly manage elderly and multimorbid patients. In this context, the index diagnosis may reflect the acute trigger for admission, but long-term prognosis appears to be more closely linked to cumulative cardiovascular and systemic vulnerability. Our results align with prior registry data demonstrating that age, organ dysfunction, and comorbidity burden outweigh diagnostic categories in predicting mortality beyond the index hospitalization.

These results align with previous research showing that the initial ACS diagnosis is less predictive of long-term mortality than underlying patient characteristics. In a prospective cohort from an Italian coronary care registry, Cavallini et al. observed that global in-hospital mortality was 7.2%. Key predictors of in-hospital death included advanced age (OR 2.00, *p* = 0.011), female sex (OR 2.18, *p* = 0.003), cardiac arrest (OR 12.21, *p* < 0.001), heart failure or cardiogenic shock (OR 9.99, *p* < 0.001), sepsis or septic shock (OR 5.54, *p* < 0.001), and acute kidney injury (OR 3.25, *p* = 0.021). Notably, a primary diagnosis of acute heart failure or a condition other than ACS or heart failure was also associated with a higher risk. During a mean follow-up of 17.4 ± 4.8 months, 96 all-cause deaths occurred among patients who survived hospitalization, corresponding to a 1-year mortality rate of 8.2%. Independent predictors of long-term mortality included age (HR 1.08, *p* < 0.001), female sex (HR 0.59, *p* = 0.022), presence of ≥3 comorbidities (HR 1.60, *p* = 0.047), acute kidney injury (HR 3.15, *p* = 0.001), inotropic support (HR 2.54, *p* = 0.002), and a primary diagnosis of acute heart failure. These findings reinforce our observation that age and comorbid burden, rather than ACS subtype, are key determinants of both early and late mortality following CICU admission. They also highlight the importance of comprehensive risk assessment that extends beyond index diagnosis and incorporates organ dysfunction, frailty, and treatment intensity [12]. Similar findings were echoed in U.S.-based data from the CRUSADE registry and other large databases, where clinical complexity outweighed diagnosis in long-term prognostic models [13,14,15].

The higher crude mortality observed among NSTEMI patients likely reflects their advanced age and greater prevalence of comorbid conditions, including prior ischemic heart disease and heart failure. In contemporary practice, NSTEMI patients admitted to the CICU often represent a selected high-risk subgroup, characterized by hemodynamic instability, recurrent ischemia, arrhythmias, or reduced left ventricular function. Therefore, the adverse long-term prognosis observed in NSTEMI may be attributable to baseline risk profile rather than ACS subtype per se. The attenuation of subtype effects after multivariable adjustment supports this interpretation. The high mortality in NSTEMI patients reflects their older age, multimorbidity, and higher rates of prior ischemia and heart failure—as confirmed in previous observational studies [16,17,18,19].

Our findings are consistent with recent large observational cohorts describing the growing proportion of elderly CICU patients. Older individuals frequently present with bradyarrhythmias, valvular disease, atrial fibrillation, and decompensated heart failure, and undergo distinct procedural strategies compared with younger patients. Across multiple datasets, advanced age has emerged as a dominant determinant of both in-hospital and long-term mortality. Together, these observations underscore the transition of the CICU from a diagnosis-driven to a complexity-driven care environment. In a recent large observational cohort from Fondazione Policlinico Universitario A. Gemelli IRCCS (Rome, Italy), which included over 2500 CICU patients from 2020 to 2024, 41.4% were aged ≥75 years. Compared to younger individuals, older patients were more frequently admitted for bradyarrhythmias, valvular disease, atrial fibrillation, and Takotsubo syndrome, while STEMI, myocarditis, and pulmonary embolism were less common admission diagnoses. Procedural strategies also diverged substantially with age: the elderly underwent fewer invasive coronary or electrophysiological interventions but more often received pacemakers, transcatheter aortic valve replacements (TAVR), and balloon valvuloplasty. Despite a similar average length of stay, patients ≥75 years experienced significantly higher CICU mortality (9.7% vs. 4.1%, *p* < 0.001), particularly in the context of acute heart failure, STEMI, NSTEMI, and cardiogenic shock, where mortality reached 53.8% in the oldest group. Follow-up analyses confirmed a marked reduction in survival among older adults across nearly all diagnostic categories. These data further support the growing recognition that elderly CICU patients represent a high-risk, heterogeneous population with distinct care needs. Their underrepresentation in clinical trials and guidelines creates uncertainty in management, underscoring the urgent need for age-adapted clinical pathways and resource planning in intensive cardiac care [20]. While STEMI may present more acutely, NSTEMI typically affects patients with diffuse vascular disease and cumulative cardiovascular burden, increasing their vulnerability to adverse events over time.

Atrial fibrillation and prior stroke emerged as particularly strong independent predictors of mortality in our cohort. Both conditions represent markers of systemic vascular disease and cumulative cardiovascular burden. These comorbidities have been consistently associated with poor outcomes in CICU cohorts. Atrial fibrillation has been linked with increased mortality post-ACS due to its associations with systemic embolism, heart failure, and treatment challenges [21,22]. Stroke, a marker of advanced vascular disease and systemic frailty, was associated with a >6-fold increased odds of death in our population—a finding echoed in prior CICU outcome studies [23].

Also, significant sex-related differences in management were observed, with women less frequently undergoing coronary angiography and PCI compared to men. This disparity is likely multifactorial. Women in our cohort were older and exhibited a higher burden of comorbidities, which may have influenced clinical decision-making and the perceived risk–benefit ratio of invasive strategies. In this context, lower rates of invasive procedures may reflect appropriate risk stratification rather than systematic undertreatment. However, the possibility of sex-related disparities in access to invasive care cannot be excluded. Previous studies have consistently reported lower rates of invasive management in women with acute coronary syndromes, even after adjustment for clinical characteristics. In our analysis, sex was not an independent predictor of long-term mortality after multivariable adjustment, suggesting that baseline vulnerability rather than sex itself was the primary determinant of outcomes. Nonetheless, the observed differences in management highlight the need for continued attention to potential inequities in care and individualized treatment decisions. Results from other studies suggest that observed sex differences in outcomes are largely mediated by age and baseline risk factors rather than intrinsic biological differences in long-term survival after ACS [24,25].

Interestingly, dementia showed a strong unadjusted association with mortality but lost statistical significance in the multivariable model. This may be likely due to the small number of affected patients and limited statistical power. In addition, dementia is closely linked with advanced age, raising the possibility of collinearity between these variables. Although cognitive impairment may represent an important dimension of patient vulnerability, the present study was not adequately powered to disentangle its independent effect from biological aging. Further studies with larger sample sizes are needed to clarify the role of cognitive decline as an independent determinant of long-term outcomes. Nevertheless, cognitive impairment remains a recognized barrier to secondary prevention, medication adherence, and long-term rehabilitation [26].

Our findings support previous calls for modern CICUs to adopt risk-based—rather than diagnosis-based—follow-up strategies. As critical care continues to expand beyond STEMI-focused care into the management of older, medically complex patients, accurate long-term prognostication will require multidimensional models that incorporate cardiovascular, neurologic, and geriatric variables. The inclusion of discharge pharmacotherapy in the multivariable model did not materially alter the observed associations, suggesting that long-term prognosis was driven predominantly by baseline clinical vulnerability rather than differences in secondary prevention. While optimal medical therapy remains a cornerstone of post-discharge management, these findings support the concept that intrinsic patient factors play a critical role in determining long-term outcomes.

Finally, In the present study, a high comorbidity burden, reflected by a CCI ≥ 5, was independently associated with increased 5-year mortality. Importantly, this association persisted after adjustment for age and key clinical variables, suggesting that cumulative disease burden captures a dimension of patient risk that is not fully explained by individual diagnoses alone. From a pathophysiological and clinical perspective, the CCI may be interpreted as a surrogate marker of overall biological vulnerability, integrating the impact of multiple coexisting conditions, systemic inflammation, and reduced physiological reserve. In this context, our findings support the concept that long-term prognosis in critically ill cardiac patients is driven not only by the index cardiovascular event but also by the broader clinical profile of the patient. Notably, while individual conditions such as prior stroke and atrial fibrillation remained significant predictors of mortality, the independent contribution of a high CCI underscores the importance of considering multimorbidity in risk stratification. This is particularly relevant in contemporary CICU populations, which are increasingly characterized by advanced age and complex comorbidity profiles. These observations are consistent with previous studies demonstrating that comorbidity indices provide incremental prognostic value beyond traditional risk factors. Taken together, our results support a shift from diagnosis-centered to patient-centered risk assessment, in which cumulative vulnerability plays a central role in determining long-term outcomes [27,28].

Importantly, this dataset contributes several novel elements to the existing literature. First, it provides complete 5-year follow-up in a contemporary, consecutive CICU cohort, extending beyond the short- and intermediate-term horizons typically reported in coronary care registries. Second, the study integrates detailed characterization of comorbidity burden, invasive management patterns, and ACS subtype within a unified multivariable framework, allowing direct comparison between diagnostic and patient-related determinants of long-term prognosis. Third, by demonstrating that ACS subtype loses prognostic significance after adjustment for age and comorbidities, our findings empirically support the conceptual shift from diagnosis-centered to vulnerability-centered risk stratification in modern CICU populations. Together, these aspects position the present analysis as a bridge between traditional coronary care research and emerging complexity-driven models of intensive cardiovascular care.

### Study Limitations

This study has several limitations. First, its retrospective, single-center design may limit generalizability to other healthcare systems with different CICU structures and admission criteria. Second, although comprehensive clinical data were collected, certain variables—such as frailty indices, socioeconomic status, and detailed discharge pharmacotherapy—were not systematically available and may have influenced long-term outcomes. Third, long-term follow-up was limited to the ACS subgroup, which may not fully represent trajectories of other CICU admission diagnoses. Finally, cause-specific mortality was not adjudicated, precluding differentiation between cardiovascular and non-cardiovascular deaths.

Additionally, an important limitation of the present study is that long-term outcome analyses were restricted to patients with ACS, which may introduce selection bias and limit generalizability to the broader CICU population. In particular, patients admitted with acute heart failure or cardiogenic shock—who represent a highly vulnerable subgroup with the highest in-hospital mortality—were not included in long-term analyses. As a result, the long-term cohort represents a selected population of survivors with relatively lower early mortality risk. This survival bias should be taken into account when interpreting the relative impact of diagnosis versus comorbidity burden. Therefore, the concept of “vulnerability” presented in this study applies primarily to patients with ACS and should not be directly extrapolated to all CICU admissions.

## 5. Conclusions

In this contemporary, real-world CICU cohort, ACS was the leading admission diagnosis. Although NSTEMI patients exhibited the highest crude 5-year mortality, differences across ACS subtypes were no longer significant after adjustment for age and comorbidities. Independent predictors of long-term mortality included advanced age, atrial fibrillation, and prior stroke. These findings indicate that underlying patient risk profile, rather than index diagnostic category, predominantly determines long-term prognosis following emergent CICU admission.

## Figures and Tables

**Table 1:** Baseline characteristics.

Variable	Total N = 1299 (%)	Females N = 511 (%)	Male N = 788 (%)	*p*-Value
Males	788 (60.7%)	0	788 (100%)	<0.0001
Age [years]	69.0 ± 14.5	72.5 ± 14.1	66.8 ± 14.2	<0.0001
Sudden cardiac arrest at admission	80 (6.1%)	25 (4.9%)	55 (7.0%)	0.156
Diabetes mellitus	354 (27.1%)	143 (28.0%)	211 (26.8%)	0.655
Arterial hypertension	949 (72.8%)	388 (75.9%)	561 (71.2%)	0.064
Coronary artery disease	578 (44.3%)	204 (39.9%)	374 (47.5%)	0.009
Chronic kidney disease	202 (15.5%)	77 (15.1%)	125 (15.9%)	0.754
Neoplasm	192 (14.7%)	89 (17.4%)	103 (13.1%)	0.037
Prior heart failure	280 (21.5%)	106 (20.7%)	174 (22.1%)	0.581
Prior CABG	76 (5.8%)	14 (2.7%)	62 (7.9%)	0.000
Prior PCI	282 (21.6%)	83 (16.2%)	199 (25.3%)	0.000
Dementia	63 (4.8%)	38 (7.4%)	25 (3.2%)	0.001
Atrial fibrillation	280 (21.5%)	132 (25.8%)	148 (18.8%)	0.003
ICD/CRTD	41 (3.1%)	8 (1.6%)	33 (4.2%)	0.009
Prior stroke	111 (8.5%)	42 (8.2%)	69 (8.8%)	0.762
Charlson Comorbidity Index	3.2 ± 1.9	4.3 ± 2.1	3.2 ± 1.8	<0.001

CABG—coronary artery bypass graft; PCI—percutaneous coronary intervention; ICD/CRTD—Implantable Cardioverter–Defibrillator/Cardiac Resynchronization Therapy with Defibrillator.

**Table 2:** Reasons for the cardiac intensive care unit admission and management.

Reason for Admission	Total N = 1299 (%)	Females N = 511 (%)	Male N = 788 (%)	*p*-Value
Acute coronary syndrome	825 (63.3%)	314 (61.4%)	511 (64.8%)	0.216
STEMI	239 (18.3%)	87 (17.0%)	152 (19.3%)	0.94
NSTEMI	396 (30.4%)	142 (27.8%)	254 (32.2%)	
UA	196 (15.0%)	88 (17.2%)	108 (13.7%)	
Cardiac arrhythmia	98 (7.5%)	40 (7.8%)	58 (7.4%)	0.748
Aortic stenosis	16 (1.2%)	11 (2.2%)	5 (0.6%)	0.020
Pulmonary embolism	65 (5.0%)	27 (5.3%)	38 (4.8%)	0.698
Pulmonary edema	44 (3.4%)	24 (4.7%)	20 (2.5%)	0.041
Myocarditis	38 (2.9%)	10 (2.0%)	28 (3.6%)	0.128
Acute or decompensated chronic HF	92 (7.1%)	40 (7.8%)	52 (6.6%)	0.439
Others	124 (9.5%)	46 (9.0%)	78 (9.9%)	0.630
In-hospital management				
Coronary angiography	870 (66.7%)	317 (62.0%)	553 (70.2%)	0.003
PCI	667 (51.2%)	211 (41.3%)	456 (57.9%)	0.000
CABG	104 (8.0%)	42 (8.2%)	62 (7.9%)	0.835

HF—heart failure; NSTEMI—non-ST elevation myocardial infarction; STEMI—ST elevation myocardial infarction; UA—unstable angina; PCI—percutaneous coronary interventions; CABG—coronary artery bypass grafting.

**Table 3:** The rates of in-hospital deaths according to admission causes.

Reason for Admission	Total Number per Reason for Admission	Deaths N (%)	Survivors N (%)	*p*-Value
Acute coronary syndrome	825	62 (7.5%)	763 (92.5%)	0.458
STEMI	239	33 (13.8%)	206 (86.2%)	0.001
NSTEMI	396	26 (6.6%)	370 (93.4%)	0.266
UA	196	5 (2.6%)	191 (97.4%)	0.001
Cardiac arrhythmia	98	5 (5.1%)	93 (94.9%)	0.336
Aortic stenosis	16	1 (6.2%)	15 (93.8%)	1.000
Pulmonary embolism	65	5 (7.7%)	60 (92.3%)	1.000
Pulmonary edema	44	7 (15.9%)	37 (84.1%)	0.079
Myocarditis	38	1 (2.6%)	37 (97.4%)	0.358
Acute or decompensated chronic heart failure	92	17 (18.5%)	75 (81.5%)	0.001
Others	124	5 (4.0%)	119 (96.0%)	0.114

NSTEMI—non-ST elevation myocardial infarction; STEMI—ST elevation myocardial infarction; UA—unstable angina.

**Table 4:** Clinical outcomes at 5-year follow-up according to ACS subtype.

Endpoint	STEMI (N = 239)	NSTEMI (N = 396)	UA (N = 196)	Total (N = 825)	*p*-Value
All-cause death	44 (18.4%)	123 (31.1%)	47 (24.0%)	214 (25.9%)	<0.001 ^1^
Unplanned cardiovascular hospitalization	62 (25.9%)	118 (29.8%)	49 (25.0%)	229 (27.8%)	0.352
Myocardial infarction	10 (4.2%)	22 (5.6%)	10 (5.1%)	42 (5.1%)	0.659
Heart failure	29 (12.1%)	63 (15.9%)	31 (15.8%)	123 (14.9%)	0.319
Stroke	7 (2.9%)	10 (2.5%)	8 (4.1%)	26 (3.2%)	0.597

^1^ Post hoc Fisher’s exact test with Bonferroni correction for all-cause death: STEMI vs. NSTEMI: *p* = 0.0003 (significant), STEMI vs. UA: *p* = 0.41 (NS), NSTEMI vs. UA: *p* = 0.22 (NS); NSTEMI—non-ST elevation myocardial infarction; STEMI—ST elevation myocardial infarction; UA—unstable angina.

**Table 5:** Adjusted hazard ratios (multivariable model) for all-cause death at 5 years in acute coronary syndrome patients.

Variable	Adjusted HR	95% CI	*p*-Value
Age (per year)	1.05	1.03–1.07	<0.001
Atrial fibrillation	1.65	1.08–2.53	0.021
Prior stroke	3.12	1.54–6.31	0.002
Dementia	1.74	0.57–5.28	0.329
Charlson Comorbidity Index ≥ 5	1.88	1.24–2.86	0.003

## Data Availability

Data are to be retrieved from the corresponding author on request.

## Supplementary Materials

The following supporting information can be downloaded at: https://www.mdpi.com/article/10.3390/life16040586/s1, Table S1: Univariable Cox analysis.

## Abbreviations

The following abbreviations are used in this manuscript:

ACS	Acute Coronary Syndrome
AF	Atrial Fibrillation
CABG	Coronary Artery Bypass Grafting
CCI	Charlson Comorbidity Index
CI	Confidence Interval
CICU	Cardiac Intensive Care Unit
CKD	Chronic Kidney Disease
CVD	Cardiovascular Disease
HR	Hazard Ratio
MI	Myocardial Infarction
NSTEMI	Non-ST-Elevation Myocardial Infarction
OR	Odds Ratio
PCI	Percutaneous Coronary Intervention
STEMI	ST-Elevation Myocardial Infarction
UA	Unstable Angina
