# Supplementary material for: Determinants of Long-Term Mortality in Patients with Acute Coronary Syndromes Requiring CICU Admission: Diagnosis Versus Vulnerability in a Contemporary Real-World Cohort
Source: Life (Basel). 2026 Apr 1;16(4):586. doi: 10.3390/life16040586 (PMC13118271; doi:10.3390/life16040586)
Supplement: Supplementary file 1 [file life-16-00586-s001.zip › life-4206624-supplementary.pdf]

Supplementary Table S1. Univariable Cox analysis

Variable	HR	95% CI	p-value
Dementia	7.27	3.01–17.52	0.000
Atrial fibrillation (AF)	3.10	2.12–4.55	0.000
Stroke (at baseline)	6.39	2.86–14.25	0.000
Myocardial infarction (MI)	3.58	1.97–6.50	0.000
LVEF < 45%	3.22	2.36–4.38	0.000
Heart failure (prior)	5.42	3.48–8.44	0.000
Cancer	3.89	2.62–5.77	0.000
Heart failure (current)	5.10	3.49–7.46	0.000
Prior ischemic heart disease (IHD)	2.51	1.85–3.40	0.000
Hypertension (HTN)	3.02	2.01–4.55	0.000
Age (per year)	1.09	1.07–1.11	0.000
Chronic kidney disease (CKD)	4.48	2.87–6.97	0.000
Conservative treatment	4.44	2.14–9.24	0.0001
Diabetes mellitus	1.92	1.40–2.64	0.0001
Coronary angiography	0.26	0.13–0.53	0.0002
Stroke (any)	2.78	1.56–4.98	0.0006
NSTEMI	1.68	1.25–2.27	0.0007
STEMI	0.56	0.40–0.80	0.0012
Prior PCI	1.62	1.15–2.29	0.0056
ICD/CRTD device	4.63	1.54–13.96	0.0065
PCI (current hospitalization)	0.67	0.47–0.96	0.0278
Prior CABG	1.69	0.93–3.08	0.0857
CABG (current)	0.55	0.12–2.57	0.4485
Unstable angina (UA)	0.87	0.60–1.26	0.4691
COVID-19 vaccination	0.81	0.42–1.56	0.526
Male sex	0.94	0.69–1.28	0.7099
Charlson Comorbidity Index			
0-2	1.0	Reference	-
3-4	1.52	1.12-2.07	0.007
≥5	2.28	1.56-3.33	<0.001
Antiplatelet drugs	0.72	0.51-1.01	0.056
Beta-blockers	0.84	0.61-1.16	0.287
Statins	0.63	0.42-0.95	0.028
ACE inhibitors/ARB	0.79	0.58-1.08	0.139
